# Biomimetic Approach for the Elaboration of Highly Hydrophobic Surfaces: Study of the Links between Morphology and Wettability

**DOI:** 10.3390/biomimetics6020038

**Published:** 2021-06-08

**Authors:** Quentin Legrand, Stephane Benayoun, Stephane Valette

**Affiliations:** Laboratoire de Tribologie et Dynamique des Systèmes, Ecole Centrale de Lyon, UMR CNRS 5513, 69130 Ecully, France; stephane.benayoun@ec-lyon.fr (S.B.); stephane.valette@ec-lyon.fr (S.V.)

**Keywords:** wetting, hydrophobic surfaces, biomimetics, replication process, multiscale roughness

## Abstract

This investigation of morphology-wetting links was performed using a biomimetic approach. Three natural leaves’ surfaces were studied: two bamboo varieties and Ginkgo Biloba. Multiscale surface topographies were analyzed by SEM observations, FFT, and Gaussian filtering. A PDMS replicating protocol of natural surfaces was proposed in order to study the purely morphological contribution to wetting. High static contact angles, close to 135${}^{\circ }$, were measured on PDMS replicated surfaces. Compared to flat PDMS, the increase in static contact angle due to purely morphological contribution was around 20${}^{\circ }$. Such an increase in contact angle was obtained despite loss of the nanometric scale during the replication process. Moreover, a significant decrease of the hysteresis contact angle was measured on PDMS replicas. The value of the contact angle hysteresis moved from 40${}^{\circ }$ for flat PDMS to less than 10${}^{\circ }$ for textured replicated surfaces. The wetting behavior of multiscale textured surfaces was then studied in the frame of the Wenzel and Cassie–Baxter models. Whereas the classical laws made it possible to describe the wetting behavior of the ginkgo biloba replications, a hierarchical model was developed to depict the wetting behavior of both bamboo species.

## 1. Introduction

Controlling the wetting properties of surfaces is an important issue for many applications. For instance, highly hydrophobic surfaces may induce anti-icing [1], self-cleaning [2], water-repellent [3,4], or wear-resistance [5] properties. According to the fundamental Young’s law of wetting on flat surfaces, many companies developed chemical surface treatments to modify the surface tension of solids and to control their wetting properties. In this sense, industrial very low adhesive surfaces are usually obtained through fluorinated compounds deposition [6,7,8]. Even though such an approach is a high-performance one in terms of watter repellency, fluorinated compounds become no more tolerable due to their toxicity for nature and humans [9]. New approaches need to be developed to obtain super-hydrophobic and water-repellent surfaces. Among them, physical surface texturing is a promising one. The Wenzel [10] and Cassie–Baxter [11] models have highlighted the role of the surface morphology onto the wetting properties of textured surfaces. These two fundamental laws have been expanded to take into account more complicated surfaces. This is the case for instance of the works of Quéré [12] and Marmur [13,14], which investigated the transition between Cassie–Baxter and Wenzel states. Bormashenko [15,16,17] and Extrand [18,19] also developed both theoretical and experimental approaches to study the conditions for a metastable Cassie–Baxter state. In wetting studies, super-hydrophobicity, defined as a state with both a static contact angle greater than 150${}^{\circ }$ and an hysteresis lower than 10${}^{\circ }$, plays a major role. The property of super-hydrophobicity is usually associated with very low adhesive surfaces. One of the most famous examples of low adhesive surface and super-hydrophobicity is the lotus effect, coming from the lotus leaf water repellency. The lotus effect has been explained for the first time by Barthlott and Neinhuis in 1997: the water repellency effect of the lotus leaf is due to its complex multiscale morphology [20]. Since this pioneering work, many super-hydrophobic natural surfaces have been identified both in the vegetable [21] and in the animal [22,23,24,25] worlds. All of these studies point out the link between the multi-scale and hierarchical structure of the surface morphology and the water repellency [26].

Theoretical approaches of hierarchical surface wetting have been developed by coupling Wenzel and Cassie–Baxter’s models as a function of scale [27]. In such approaches, the lotus leaf wetting may be depicted by a purely Cassie–Baxter state on every topogaphical scale present on the leaf. On the contrary, the rose petal wetting behavior presents a Cassie–Baxter state on nanometric scales whereas a Wenzel state may be proposed to depict the wetting behavior at the micrometric scale [28]. Such an hybrid wetting configuration of the rose petal is associated with a large static contact angle (around 150${}^{\circ }$) and a large hysteresis of contact angle (180${}^{\circ }$). Such a configuration leads to super-adhesive property of water onto rose petals despite its large static contact angle.

These amazing properties of natural surfaces are at the basis of the biomimetic approach currently under development both in academia and in the industry. The manufacturing of such hierarchical surfaces inspired from nature is performed through material removal processes such as photolithography [29] or laser ablation [30,31], through material deformation processes such as micro-knurling [32], or through material deposition [33,34]. Within these various technical processes of surface manufacturing, the elaborated surfaces are usually less complex than natural ones. Moreover, superimposed to physical texturing of the surface, the processes of surface texturing may also involve chemical modifications. To study the purely morphological-wetting links, some replication processes have been developed. Polymer injection [35,36,37] and PDMS replication [38,39,40] are good candidates to perform chemically stable replications of textured surfaces. The complexity of multiscale natural surfaces makes their analysis in terms of wetting very intricate. To propose an interpretation of the wetting contribution on such surfaces, each relevant scale has to be identified. If many inputs have been proposed in literature on human-made hierarchical surfaces [30,41], this point has been less intensively studied in the cases of natural surfaces [34,39].

In this work, three natural surfaces of plant leaves are studied: two bamboo leaves and the Gingko Biloba leaf. A specific PDMS replication protocole is proposed. At first, the topography of the surfaces is analyzed through a coupled approach: SEM observations and spatial filtering analysis from optical interferometric measurements. Second, wetting properties analysis of surfaces is performed through static and dynamical contact angles measurements. Topographical and wetting analyses are both performed on natural leaves and on replicas. Finally, using a hierarchical approach, the relationship between multiscale topography and wetting behavior is proposed.

## 2. Materials and Methods

### 2.1. Leaves

Three natural surfaces of plant leaves are studied: two different varieties of bamboo and the Ginkgo Biloba. The Ginkgo Biloba leaf has been chosen for its well-kown water-repellent properties as studied by Neinhuis and Barthlott [20]. Bamboo leaves have been chosen due to the hydrophobicity of the lower surface of their leaves, with the upper surface being hydrophilic.

**Figure 1:**
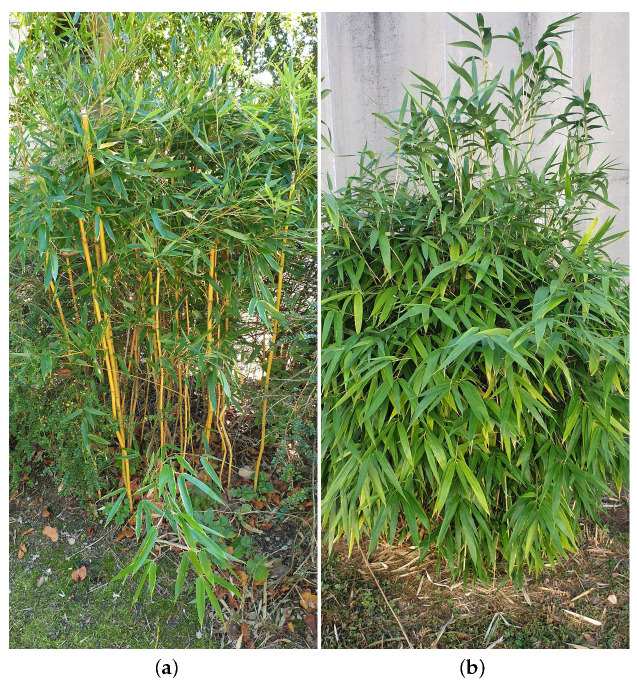
Photographs of the bamboo varieties studied: (**a**) Phyllostachys Aureosulcata, (**b**) Sasa Palmata.

The first bamboo variety studied is the *Phyllostachys Aureosulcata Aureocaulis* (Figure 1a). The second type of bamboo studied is very different from the first one in terms of leaf size. Its very large leaves as well as the characteristics of its canes and its overall appearance suggest that the second bamboo variety belongs to the Sasa Palmata family (Figure 1b). However, if the first type of bamboo is perfectly identified, there is no certainty for the second one. The great plurality of bamboo types and subtypes does not allow us, without further information, to affirm the affiliation of the second variety.

As living systems, the delay between harvesting of the leaves and their analyses is one prime interest. It has to be known and fixed. For bamboo leaves, all experiments are performed a few minutes after harvesting. For Gingko Biloba leaves, the delay between harvesting and analyses is around 1 h.

### 2.2. PDMS Replicas

From the three natural surfaces, three PDMS replicas are obtained by a replication protocol. This replication protocol is a two-step process: first, a complementary negative replica of the original surface is made. Then, the negative replica is used as a mold to obtain a positive replica of the original surface.

The replication protocol is performed with the polydimethylsiloxane (PDMS) Sylgard 184 reference in a 10:1 weight ratio with the curing agent [39,42]. To mix the silicone elastomere and the curing agent, a centrifuge is used at 3000 rpm for 3 min. The mixed PDMS is deposited onto the plant surface to be replicated and then placed in a vacuum chamber for 40 min to maximize impregnation of the PDMS in the surface texturing. After one hour of heating at 80 °C, the PDMS is fully cured and can be unmoulded to obtain the negative replica.

To obtain the positive replica, the same protocol is used with the negative replica previously made as the original surface. However, to limit PDMS/PDMS adhesion, a coating of a few nanometers of gold is made with a vacuum sputtering metallizer. This step is traditionally carried out with a fluorine treatment [39,43]. However, fluorine treatments have strong impacts on superhydrophobic properties. Therefore, in order to limit the contamination of positive replicas, gold metallization is preferred to fluorine treatment. Once this step is complete, it is possible to reproduce the previous steps to obtain a positive replica of the original natural surface.

**Figure 2:**
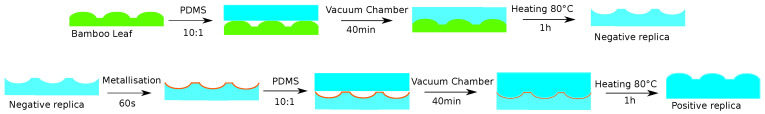
Schematic representation of the fluor-free PDMS-replication protocol.

The different steps of this PDMS-replication protocol are presented in the Figure 2.

### 2.3. Topographic Characterization

The morphologies were observed with a scanning electron microscope (MIRA3 Tescan). These observations were carried out with a secondary electron detector to reveal the topographic contrast.The various patterns constituting the topography of the surfaces are thus described. The in-plane contributions of the patterns (width and periodicity) can be measured precisely.

The characterization of the morphology by SEM was completed using a quantitative analysis based on optical inteferometric measurements. These measurements were carried out in VSI mode (Vertical Scanning Interferometry), with green light (515 nm wavelength). The analyses of interferometric measurements were performed with the MountainsMap software (DigitalSurf) using the ISO-25178 roughness standard [44].

**Figure 3:**
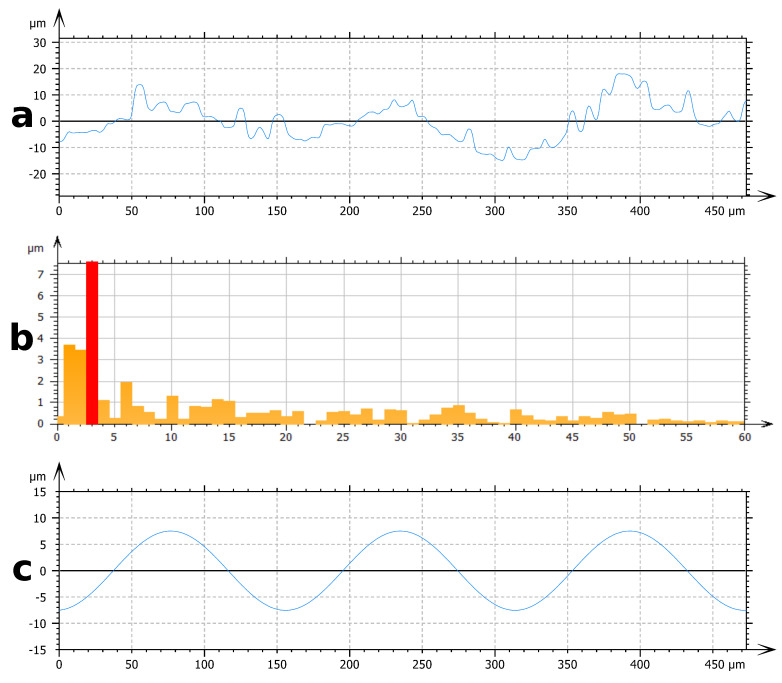
Example of frequency filtering by FFT: (**a**) unprocessed profile of Phyllostachys Bamboo leaf, (**b**) frequency spectrum with in red the selected frequency, and (**c**) filtered profile.

**Figure 4:**
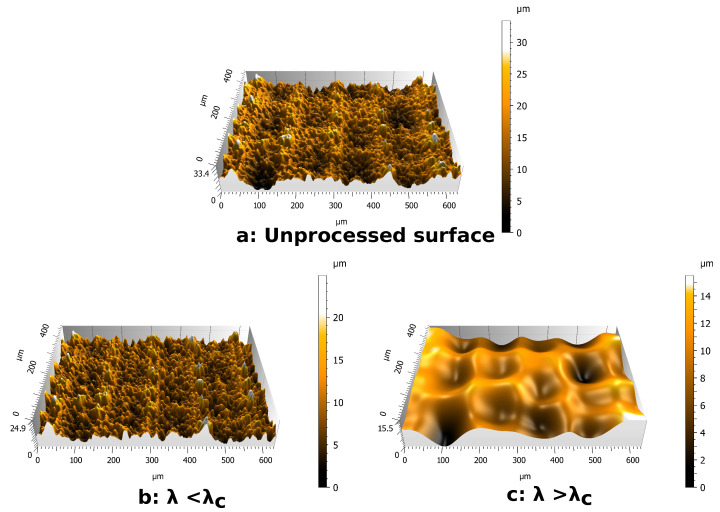
Example of Gaussian filtering, with a cut-off of 120 μm, on Phyllostachys bamboo leaf: (**a**) unprocessed surface, (**b**) filtered surface for small scales, and (**c**) filtered surface for high scales.

To overcome the difficulty of topographic analysis of natural leaves due to their hierarchical structure, two spatial filtering analysis methodologies are proposed. They enable the description of each topographical scale separately:

- The first filtering method involves Fast Fourier Transform (FFT). This method is used to characterize the range of periodical structures. FFT is performed on an experimental profile (Figure 3a) and provides a frequency spectrum (Figure 3b). Due to the multi-scale nature of these surfaces, many signals are present on the frequency spectrum. Frequencies corresponding to the periods identified by the SEM observation are isolated. These frequencies correspond to important signals on the frequency spectrum. By inverse FFT, the selected frequency profile is obtained (Figure 3c). The filtered profile is used to measure the height of the texture associated with each periodic roughness scale.
- The second filtering process involves Gaussian filters [30]. Gaussian filters are configured by a cut-off wavelength $\lambda_{c}$. By applying a Gaussian filter to a surface, two surfaces are obtained (Figure 4). The first one contains the wavelengths lower than the cut-off value (Figure 4b). The second one contains the wavelengths higher than the cut-off value (Figure 4c). For a wavelength equal to the cut-off value, 50% of its amplitude is transmitted to each of the surfaces produced by filtering. Due to this property of Gaussian filters, it is necessary to choose a cut-off wavelength far away from the wavelengths of the scales to be separated. The choice of the cut-off value for each surface studied is based on SEM observation. This method is used to separate the scales of large period versus small period profiles.

In addition, classical roughness parameters are measured on the raw interferometric images as well as on the surfaces obtained by Gaussian filtering. The parameters calculated from the interferometric measurements are arithmetic mean height $S_{a}$ (1), root mean square height $S_{q}$ (2), and developed interfacial area ratio $S_{dr}$ (3).

(1) $$S_{a}=\frac{1}{A}\underset{A}{\int \;\;\;\int }\left|z\left(x,y\right)\right|dxdy$$

(2) $$S_{q}=\sqrt{\frac{1}{A}\underset{A}{\int \;\;\;\int }z{\left(x,y\right)}^{2}dxdy}$$

(3) $$S_{dr}=\frac{1}{A}\underset{A}{\int \;\;\;\int }\left(\sqrt{\left(1+{\left(\frac{\partial z(x,y)}{\partial x}\right)}^{2}+{\left(\frac{\partial z(x,y)}{\partial y}\right)}^{2}\right)}-1\right)dxdy$$

The $S_{dr}$ measurement is used to determine the Wenzel parameter, *r* [30]. These two parameters are linked by the follows relation (4):

(4) $$r=S_{dr}+1$$

The measurement of the $S_{dr}$ parameter on surfaces obtained by Gaussian filtering enables the evaluation of roughness with large and small topographical scales.

### 2.4. Wettability

The wettability of the surfaces was quantified using a DSA 30 goniometer (Kruss). Static apparent contact angle measurements [45,46] were performed with droplets of distilled water of 3 μL. The drop was deposited using a teflon needle to limit needle adhesion. Measurements were performed using Drop Shape Analysis software and tangent-2 method [47]. For each surfaces studied, five droplets were deposited to ensure the repeatability of the measurement.

Dynamic measurements were performed by injection/absorption method. From a 3 μL droplet, the volume increases by 7 μL with an injection velocity of 10 μL/min. Then, the 10 μL droplet is absorbed with same velocity as for injection. The base diameter of the droplet is measured during the variation of volume, and it is used to detect advancing and receding of the droplet. The apparent contact angle is measured with the tangent-2 method to obtain advancing contact angle $\theta_{a}$ and receding contact angle $\theta_{r}$. For each surface, five dynamic contact angle measurements were performed.

When the texture is anisotropic, the anisotropy of the wetting properties was quantified by measuring the contact angles in two orthogonal directions: longitudinal direction (observation perpendicular to the grooves) and transversal direction (observation parallel to the grooves).

## 3. Results

### 3.1. Topography of Natural Surfaces

#### 3.1.1. Phyllostachys and Sasa Leaves

**Figure 5:**
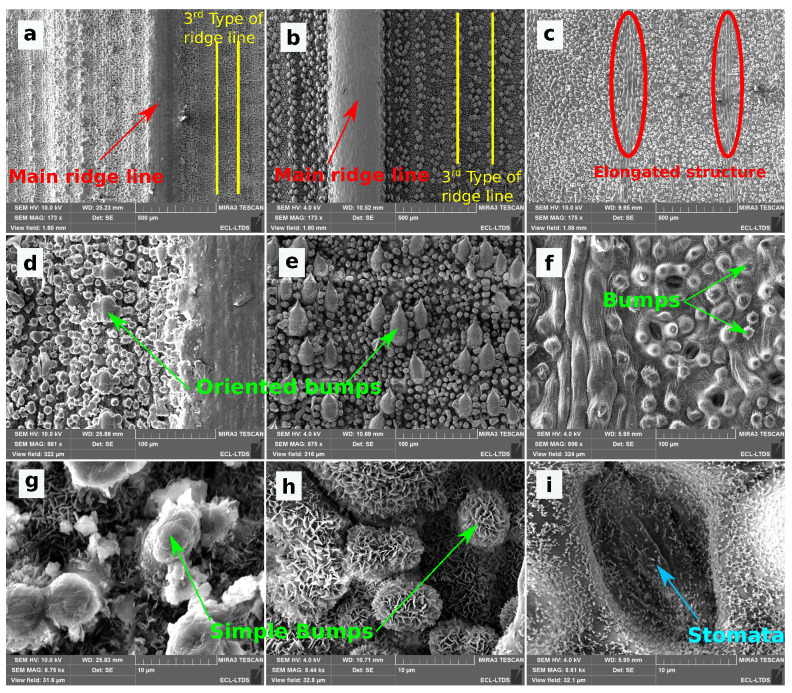
SEM images of leaves studied at different scales: (**a**,**d**,**g**) Phyllostachys, (**b**,**e**,**h**) Sasa, and (**c**,**f**,**i**) Ginkgo.

**Figure 6:**
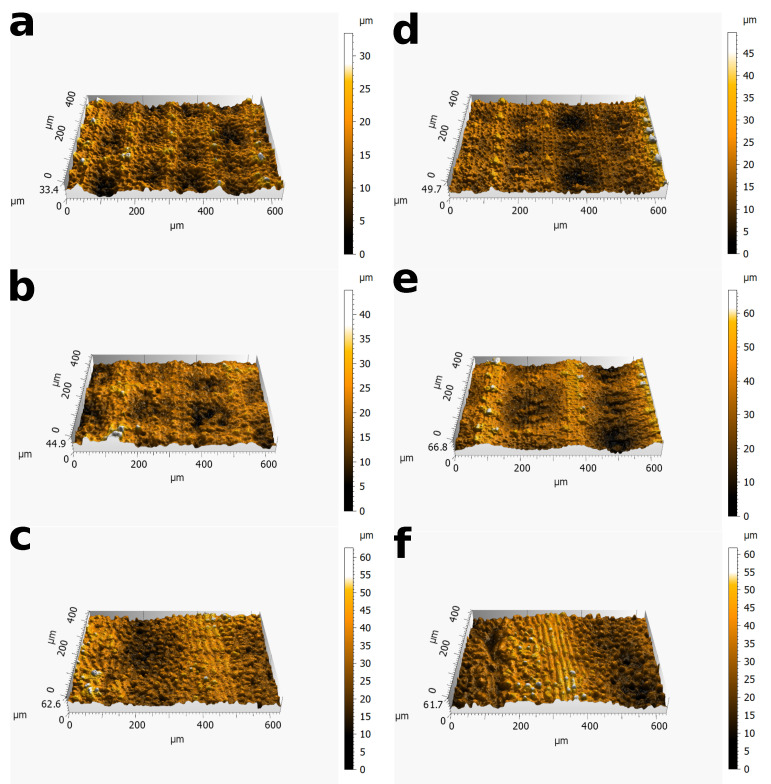
Interferometric images of texture: (**a**–**c**) Phyllostachys, Sasa, and Ginkgo, respectively; (**d**–**f**) replicas of Phyllostachys, Sasa, and Ginkgo, respectively.

Morphological and topographical characterizations of the Phyllostachys and Sasa leaves are presented, respectively, in Figure 5 and Figure 6.

The textures of both bamboo types can be classified into three categories: ridge lines, bumps, and epicuticular wax. The main ridge line is the extension of the stem of the leaf. This main ridge line is visible in Figure 5a,b. The height and width of this main ridge line are 300 μm and 600 μm on Phyllostachys. On Sasa, these dimensions are 400 μm and 700 μm. A second type of ridge line is observed. Its periodicity is in the order of millimiter for both species. Due to this large value of period, this second type of ridge line is not obvious on the SEM images. Finally, a third type of ridge line may be observed on Figure 5a,b. These ridge lines form a grid that is not observable on the SEM images but that is visible on interferometric measurements on Figure 6a,b.

The ridge lines are covered with bumps. Two different bumps are identified: oriented bumps and simple bumps. Oriented bumps are observable in Figure 5d,e. These bumps end in a spike that points towards the ending of the bamboo leaf. They are located at the peak and valley of the third-type of ridge line. There are more numerous on the Sasa leaf and arranged in packages of three to four bumps. The simple bumps are distributed in a disordered pattern. They are shown in Figure 5g,h. Simple bumps present smaller dimensions than the oriented bumps. In addition, the dimension of the simple bumps is much more scattered than for other structures.

A last roughness scale is observable on the bamboo leaves and corresponds to the presence of an epicuticular wax. This wax is superimposed on the textures already presented and adds a nanometric scale to these surfaces. This wax can be seen in Figure 5g,h.

The orders of magnitude of the dimensions of these objects are summarized in the Table 1. The period and widths of these objects are measured from SEM images and topographic measurements. The height of these scales is obtained by FFT analysis of these surfaces.

Both bamboo species possess the same pattern. However, the dimensions of these topographies are different between Phyllostachys and Sasa. The period of the third-type of ridge line measures 160 μm on the Phyllostachys, while it is evaluated at 300 μm on the Sasa. The oriented bumps are almost three times larger on the Sasa, and they are much more numerous. These differences could lead to differences in wetting properties. In addition, ridge lines and oriented bumps bring an anistropy on the topographies. This anisotropy could lead to anisotropic wetting properties.

#### 3.1.2. Ginkgo Biloba

The morphology and the topography of the ginkgo biloba leaf are presented in Figure 5 and Figure 6. The topography of ginkgo biloba is much less complex than the topography of both bamboo types.

Two areas of different textures can be observed on SEM images and interferometric measurements. The first one, which is very predominant, is composed of bumps with a diameter of 15–20 μm. These bumps can be seen on SEM images (Figure 5f). The second zone is localized. It is composed of an elongated structure that is 100 μm long and 20 μm wide. These structures are juxtaposed and form 80 μm wide lines. These lines converge towards the point of binding between the stem and the leaf. These structures are shown in the Figure 5c.

In addition, the structures of ginkgo biloba are covered by an epicuticular wax visible on the SEM images (Figure 5i). However, this wax presents a different aspect from that observed on the bamboo leaves. It is indeed more filamentary and less covering.

Among the bumps, it is also possible to observe structures associated with the biological behavior of the ginkgo biloba leaf: the stomata. The stomata is a structure that ensures gas exchange between the leaf and its environment. These structures, which are observed on many plants, are shown in Figure 5i.

### 3.2. Topography of PDMS Replicas

**Figure 7:**
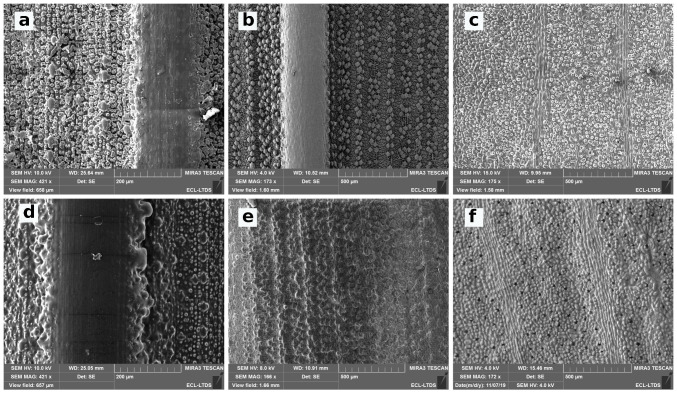
SEM images of (**a**) Phyllostachys, (**b**) Sasa, and (**c**) Ginkgo Biloba leaves and (**d**–**f**) their respective PDMS replica.

**Figure 8:**
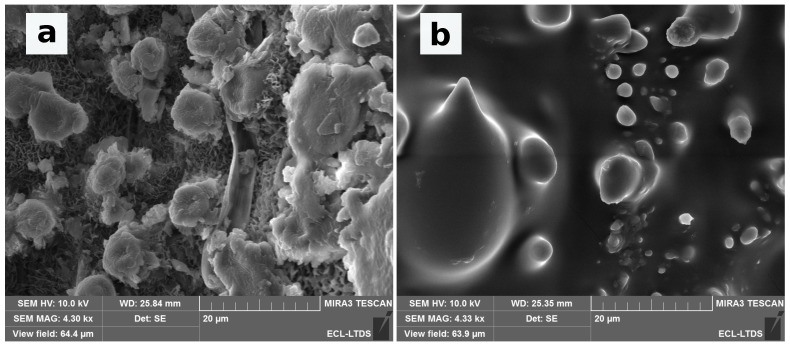
SEM images of (**a**) Phyllostachys leaves and (**b**) Phyllostachys replicas.

The texture of the PDMS replicas is presented in Figure 7 and Figure 8. The Figure 7 indicates that the micrometric structures of PDMS replicas are closed to the original leaves. However, the nanometric scale brought by the epicuticular wax is not observable on the Phyllostachys replica shown in Figure 8. The same observation is made for the replica of Sasa and Ginkgo. The disappearance of the epicuticular wax occurs from the first step of the replication protocol. Indeed, SEM observations on negative replicas show that the epicuticular wax is not replicated by the first step. This disappearance is not surprising and has already been observed in other PDMS replication studies [39]. The most probable hypothesis of this disappearance is based on the chemical properties of the epicuticular wax. Epicuticular wax is composed of aliphatic, cyclic, and sterol compounds [39]. These compounds are not resistant to the temperature used for the replication protocol. This disappearance could also be related to the physicochemical properties of PDMS [48].

Leaves and replicas are also compared by interferometric measurements (Figure 6). On bamboo surfaces, these interferometric measurements are processed by Gaussian filtering in order to dissociate the large scales associated with the ridge lines and the small scales associated with the bumps. The cut-off value chosen for Phyllostachys surfaces is $\lambda_{c}=120$ μm. On Sasa surfaces, the cutoff value chosen is $\lambda_{c}=225$ μm . These wavelengths are between the wavelength associated with the third type of ridge line and the wavelength of the oriented bumps (Table 1). These cutoff wavelengths allow for efficient dissociation of these scales, as shown in Figure 4.

On ginkgo surfaces, only one micrometric pattern is present; consequently, no filtering approach is involved on this type of natural surface.

The roughness parameters $S_{a}$, $S_{q}$, and $S_{dr}$ are calculated on interferometric measurements in order to compare replicas and leaves. These parameters are summarized in the Table 2.

The results on $S_{a}$ and $S_{q}$ show differences between the leaves and the replicas. Indeed, the measurements on the replicas are systematically larger than the values obtained on the natural leaves, with a maximum evolution of 205% on ridge lines $S_{a}$ measurements for Sasa surfaces. The $S_{dr}$ also shows differences between the replicas and the leaves. In the case of bamboo types, these differences are even more pronounced: on bamboo replicas, the Sdr is two times larger than the value on the natural leaves. These large differences may be due to the scattering of the roughness parameters from leaf to leaf. Indeed, the PDMS replication protocol needs two leaves to be implemented: one leaf for direct characterizations and one leaf to be impressed with PDMS. Even if specific care is taken for leaf harvesting, the topography may vary from one leaf to another [49]. The differences in roughness parameters may also be due to the disappearance of the epicuticular wax. Concerning the ginkgo biloba leaf, this large difference in Sdr parameter is not observed.

The $S_{a}$ and $S_{q}$ values on Sasa surfaces associated with bumps are higher than the values measured on Phyllostachys surfaces. This observation indicates a greater variation in z-position on Sasa surfaces ((1) and (2)). It corresponds to a greater variation in texture heights on these surfaces, an observation consistent with the measurements presented in Table 1.

A last observation concerns the comparison of $S_{dr}$ values between ridge lines and bumps. $S_{dr}$ of ridge lines is around 1%, whereas it reaches 30% on bumps. These difference may be explained by the high sensibilites of the $S_{dr}$ to the small-size pattern [30]. For the same reason, $S_{dr}$ is higher on Phyllostachys bumps than on Sasa bumps.

**Figure 9:**
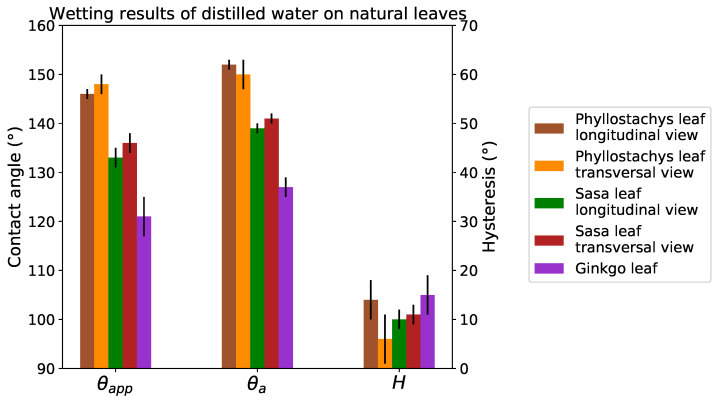
Wetting results on leaves.

The wetting experiments carried out on plant surfaces show a high hydrophobicity of these surfaces (Figure 9). Both species of bamboo present static contact angles greater than 130${}^{\circ }$. The contact angle even reaches 145${}^{\circ }$ in the case of the Phyllostachys species. On the Ginkgo leaf, the contact angle is 120${}^{\circ }$. This result differs from the experiments carried out by Neinhuis and Barthlott [20], who measured an angle of 160${}^{\circ }$. This difference can be explained by a longer time between the harvesting of the leaf and the wetting experiments. From the point of view of the wetting dynamics, the hysteresis of these plants is always less than 15${}^{\circ }$. These low hysteresis values demonstrate the non-adhesive properties of these plants and their strong abilities for water repellency.

### 3.3. Wetting Results

**Figure 10:**
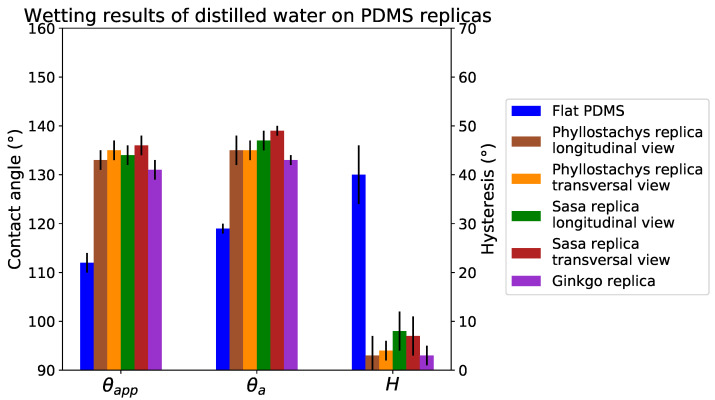
Wetting results on flat PDMS and replicas.

**Figure 11:**
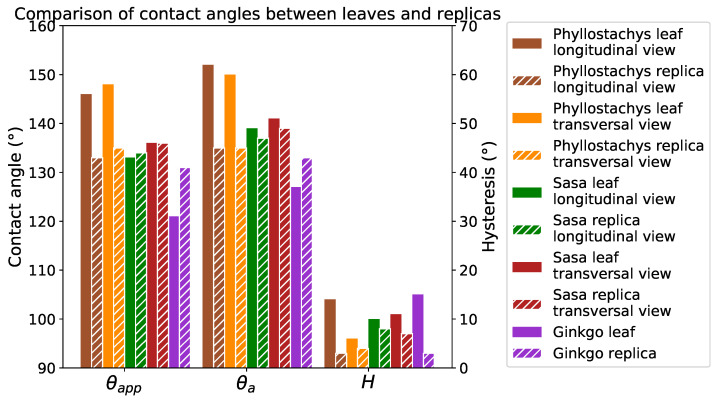
Comparison of contact angles between leaves and replicas in each direction.

The positive replicas of these plants present static contact angles greater than 130${}^{\circ }$ (Figure 10). In addition, hysteresis on replicas is always lower than 10${}^{\circ }$. Despite some differences, the replicas present wetting properties close to the original leaves (Figure 11). These differences are due to a difference in the chemistry of the materials and the absence of the roughness provided by the epicuticular wax. However, as the overall behavior of the leaves is close to replication (especially for hysteresis), it is reasonable to suppose that their wetting configurations are comparable.

The contact angles on the replicas compared to the flat PDMS indicate a very high texture contribution to the hydrophobicity of these surfaces (Figure 10). The static contact angle on the replicas is 20${}^{\circ }$ greater than on the flat PDMS. In addition, the 40${}^{\circ }$ for the hysteresis on flat PDMS is lowered to 10${}^{\circ }$ on the replicas. Due to the anisotropic nature of bamboo topographies, contact angles are measured in two directions on natural leaves and on the replicas. Despite this strong topographical anisotropy, a tiny anisotropy in wetting properties is observed.

The positive replicas present very close contact angles despite topographic differences. This observation does not ensure that the wetting configurations of these surfaces are identical. However considering the very low hysteresis values (<$10^{\circ }$), the wetting state of these replicas and leaves seems closer to a Cassie–Baxter state.

## 4. Discussion

The measurements of contact angles on the replicas show high hydrophobicity due to the texture. The very low value of hysteresis of these surfaces is even more impressive: it reveals low adhesive properties of textured PDMS replicas. Such a behavior is generally characteristic of a wetting state similar to a Cassie–Baxter type. On hierarchical surfaces, several wetting configurations involve a Cassie–Baxter state associated with one or more scales. The hierarchical approach [27] to wetting allows for the calculation of an apparent contact angle for each of these configurations. This apparent contact angle is related to the Wenzel parameter *r* for the Wenzel state (5), to the solid fraction ϕ for the Cassie–Baxter state (6) at each involved scale, and to $\theta_{PDMS}$ the apparent contact angle on flat PDMS.

(5) $$cos\theta_{W}=rcos\theta_{PDMS}$$

(6) $$cos\theta_{CB}=\phi_{S}\left(cos\theta_{PDMS}+1\right)-1$$

On bamboo leaf replicas, the scales considered for hierarchical wetting approach are the third-type of ridge line, the oriented bumps, and the simple bumps. The other ridge lines present a periodicity too close to the droplet size, so they do not contribute to wetting [50]. Indeed, the maximum base diameter obtained on the replicas is 1400 μm (Ginkgo replica). On the bamboo replicas, the other ridge lines have a minimum periodicity of 1200 μm. Therefore, they are not relevant.

The apparent contact angle on the surfaces depends on the roughness of each involved scale. This roughness is estimated by measuring the $S_{dr}$ (Table 2) according to Equation (4). The Gaussian filtering approach thus makes it possible to establish a roughness for each roughness scale. These roughnesses are summarized in Table 3. However, single bumps and oriented bumps cannot be separated by a Gaussian filtering approach. It is therefore simply assumed that their roughness is less than the total roughness of the bumps.

The solid fractions are estimated by a simplification of the involved textures. The textures are approximated to square textures of same dimensions. The simple bumps are assumed for the calculation to be uniformly distributed. The ridge lines are approximated as square ridge lines, and the bumps are approximated as square pillars. On the Sasa replica, the oriented bumps are arranged in groups of three to four bumps. These groups of bumps are simplified into rectangular pillars with a width of one bump in the longitudinal view and a width of three bumps in the transversal view. The solid fraction of each of the scales concerned is reported in Table 3.

**Figure 12:**
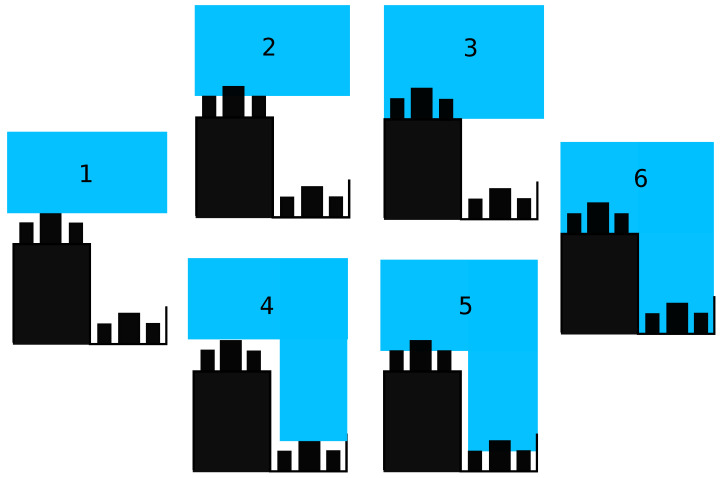
Schematic representation of the six wetting configurations investigated.

The Ginkgo replica loses its hierarchical character with the disappearance of the epicutilar wax. Only two configurations are thus envisaged, the pure state of Cassie–Baxter and the pure state of Wenzel. For bamboo replicas, six wetting configurations are considered. These configurations are schematized on the Figure 12. The description as well as the expression of the apparent contact angle of these configurations is as follows:

1. The pure Cassie–Baxter state: oriented bumps and ridge lines wet in the Cassie–Baxter condition. The apparent contact angle is $cos\theta_{app}=\left\{\phi_{RL}\times \phi_{OB}\right\}\times \left(cos\theta_{PDMS}+1\right)-1$.
2. The second pure Cassie–Baxter state: all scales wet in the Cassie–Baxter condition. The contact angle becomes $cos\theta_{app}=\left\{\phi_{RL}\times \left(\phi_{OB}+\phi_{SB}\right)\right\}\times \left(cos\theta_{PDMS}+1\right)-1$
3. Mixed state: ridge lines wet in Cassie–Baxter and the bumps wet in Wenzel. The expression of its apparent contact angle is $cos\theta_{app}=\phi_{RL}\times \left(\left[r_{OB}+r_{SB}\right]\times cos\theta_{PDMS}+1\right)-1$.
4. Mixed state: ridge lines wet in Wenzel and the oriented bumps wet in teh Cassie–Baxter state. The predicted contact angle for this configuration is $cos\theta_{app}=r_{RL}\times \left(\phi_{OB}\times \left(cos\theta_{PDMS}+1\right)-1\right)$.
5. Mixed state: ridge lines wet in Wenzel state and all of the bumps wet in the Cassie–Baxter state. The predicted contact angle is $cos\theta_{app}=r_{RL}\left(\left\{\phi_{OB}+\phi_{SB}\right\}\times \left(cos\theta_{PDMS}+1\right)-1\right)$.
6. The pure Wenzel state: all scales wet in the Wenzel condition. The apparent contact angle becomes $cos\theta_{app}=\left[r_{RL}\times \left(r_{OB}+r_{SB}\right)\right]\times cos\theta_{PDMS}$.

The apparent contact angles of these configurations are summarized in Table 4. By comparison of the predicted contact angles with the experimental ones, it is possible to deduce the wetting configuration of the surfaces.

For the Phyllostachys replica, the hypothesis of configuration 6, the pure Wenzel state, is immediately eliminated due to a large difference between the experimental angle and the predicted angle. This is consistent with the low hysteresis measurements. The hypothesis of a pure Cassie–Baxter on oriented plot (configuration 1) also seems unlikely, with a predicted angle of 166${}^{\circ }$. Similarly, configurations 2 and 4 predict an angle greater than the experimental angle. Thus, these configurations are not relevant. Finally, considering the predicted contact angles, the most relevant configurations are configurations 3 and 5. The predicted contact angle for configuration 5 is 142${}^{\circ }$, a value close to the experimental measurement of 135${}^{\circ }$. The configuration 3 predicts an angle of 133${}^{\circ }$, which is also very close to the static angle measurement. However, on these surfaces, the hysteresis is less than 10${}^{\circ }$. This observation is not very compatible with configuration 3. Indeed, the Wenzel contact on the bumps brings a large area of solid–liquid contact, which is associated with a strong hysteresis according to Dubov’s work [51,52]. Therefore, the most probable configuration for Phyllostachys surfaces is configuration 5.

The same approach is developed for the Sasa replica. The hypothesis of a pure Wenzel (configuration 6) wetting is also doubtful. The predicted contact angle is much lower than the experimental value. Configurations 4 and 5 also do not explain the experimentally measured contact angles. The predicted angles for configurations 1, 2, and 3 are much more consistent with the measured values. Among these three configurations, configuration 3 predicts a Wenzel-type wetting for bumps. However, for Phyllostachys surfaces, this configuration is not revelant for the hysteresis contact angle value. The probable configuration for Sasa surfaces is therefore among configurations 1 and 2.

For the replica of Ginkgo Biloba, the pure Cassie–Baxter state (configuration 1) predicts 144${}^{\circ }$ for the contact angle, while 121${}^{\circ }$ is predicted for the Wenzel state. Compared to the 131${}^{\circ }$ obtained experimentally, none of these configurations seems possible. However, the hysteresis of this replica is also less than 10${}^{\circ }$. The pure Wenzel state cannot explain this experimental value. Thus, the wetting state of this replica is probably the mixed state between the Cassie–Baxter state and the Wenzel state described by Marmur [13].

From the point of view of contact angles and hysteresis, Phyllostachys surfaces are wet in configuration 5 and Sasa surfaces are wet in configurations 1 or 2. These supposed configurations are consistent with the other observations on the experimental measurements. The experimental values show that the hysteresis is greater on the Sasa replica than on the Phyllostachys replica. According to Dubov’s work [51,52], the hysteresis would be related to the length of the solid–liquid contact. With the size of the bumps on the Sasa surface being larger, it is consistent that the hysteresis is higher.

Another experimental observation is that texture anisotropy does not lead to anisotropy of the wettability properties. Texture anisotropy is mainly provided by the second type of ridge line and by the oriented bumps. The second type of ridge line does not contribute to the wetting: it does not bring anisotropy. Concerning the oriented bumps, in the case of Phyllostachys, it contributes less to the wetting than the simple bumps, which are much more numerous. The observation of an isotropy of the wetting properties is therefore not surprising. In the case of Sasa, configuration 1 depends a lot on the oriented bumps. However, configuration 2 depends on all of the bumps. Configuration 2 is much less anisotropic than configuration 1. Configuration 2 is therefore more relevant for the replica of Sasa, with regard to the spreading anisotropy.

Thus, it is interesting to note that, despite the similarity of patterns on the bamboo topographies, the differences in dimensions result in two different wetting configurations.

## 5. Conclusions

Natural surfaces provide very complex and often hierarchical topographies, which gives them remarkable wetting properties. These complex topographies as well as the chemistry of natural surfaces make the study of the topography-wetting link very complicated. In this study, the description of three plant topographies was carried out by coupling SEM observations and interferometric measurements. An FFT analysis allowed us to access the height of these textures. In addition, through Gaussian filtering approach, the roughness parameters associated with the different scales were determined.

Using a PDMS replication process, these topographies were reproduced on surfaces for which the chemistry was controlled. The efficiency of this replication was proven by demonstrating that the micrometric structures of these replications were similar to those observed on the original leaves. However, it was also shown that the protocol is not effective in replicating the nanometric structure of these plants.

An experimental study of the contact angles of the leaves and the replicas was carried out. The measured contact angles as well as the hysteresis showed that the plants studied presented high hydrophobic properties. These hydrophobic properties were well transferred to the PDMS, since 110${}^{\circ }$ on flat PDMS became greater than 130${}^{\circ }$ on the replicas. Still better, the 40${}^{\circ }$ hysteresis was reduced to less than 10${}^{\circ }$ on all the replicas. The contact angles between the leaves and the replicas showed some differences due to a difference in chemistry as well as the loss of the nanoscale roughness scale. However, a similar wetting behavior was observed on the replicas and on the leaves. Very low hysteresis was measured on the surfaces. The PDMS replication protocol thus allowed for the realization of controlled chemistry surfaces with topographic and wetting properties close to the original plants.

The link between the topography and wetting properties of these surfaces was thus studied through a multi-scale approach considering several wetting configurations. The topographic parameters related to the hierarchical wetting model were determined on the one hand through $S_{dr}$ measurements for roughness and on the other hand by simplifying the identified textures in order to determine a solid fraction. Several different contact angles were predicted and compared to the experimental values in order to identify the wetting configuration of each surface. It was thus shown that, despite similarities in pattern, Phyllostachys surfaces were wet in a different configuration than the Sasa surfaces. Indeed it was identified that Sasa bamboo was wet in a Cassie–Baxter state with respect to all of these micrometer scales while Phyllostachys bamboo is in a mixed state coupling the wetting of Cassie–Baxter and Wenzel. Ginkgo replica has lost its hierarchical aspect during the replication process, so the hierarchical approach used cannot be applied to it. Nevertheless, the angles predicted for the pure Cassie–Baxter and pure Wenzel configurations seem to indicate a state of mixed wetting for Ginkgo Biloba surfaces.

This work could however be extended by realizing a nanometric texture superimposed on the replicated micrometric structures. This would make it possible to study the influence of the epicuticular wax that disappeared during the replication protocol. This nanometric scale could in particular make it possible to reach a superhydrophobic behavior on polymeric surfaces.

## Figures and Tables

**Table 1:** Summary of the different textures on the surface of bamboo leaves.

Type of Texture	Phyllostachys			Sasa		
	**Period**	**Width**	**Height**	**Period**	**Width**	**Height**
	**(μm)**	**(μm)**	**(μm)**	**(μm)**	**(μm)**	**(μm)**
Main ridge line	none	600 ± 26	300 ± 11	none	700 ± 34	400 ± 10
Second type of ridge line	1200 ± 70	-	10 ± 1	2000 ± 101	-	40 ± 5
Third type of ridge line	160 ± 5	60 ± 3	15 ± 3	300 ± 12	100 ± 7	15 ± 2
Oriented bumps (main ridge line direction)	60 ± 3	20 ± 2	15 ± 2	70 ± 4	60 ± 8	30 ± 1
Oriented bumps (orthogonal direction)	80 ± 7	15 ± 1	15 ± 2	150 ± 9	40 ± 3	30 ± 1
Simple Bumps	9 ± 6	3 ± 4	3 ± 2	11 ± 5	10 ± 4	7 ± 3

**Table 2:** Topographic parameters measured on natural and replicated surfaces, based on interferometric measurements at ×10 magnification. The standart deviations associated with the $S_{a}$ and $S_{q}$ parameters are the maximum values of standard deviation for these parameters.

Surface	Pattern	Type	$S_{a}$ (μm) ± 0.7	$S_{q}$ (μm) ± 0.7	$S_{\mathrm{dr}}$ (%)
Phyllostachys ($\lambda_{c}$ = 120 μm)	ridge lines	Leaf	2.1	2.6	0.33 ± 0.02
		Replica	3.5	4.7	0.55 ± 0.15
	bumps	Leaf	2.5	3.2	15.8 ± 0.83
		Replica	3.2	4.2	36.1 ± 0.70
Sasa ($\lambda_{c}$ = 225 μm)	ridge lines	Leaf	1.7	2.2	0.12 ± 0.02
		Replica	5.2	6.2	0.74 ± 0.18
	bumps	Leaf	3.2	4.0	12.3 ± 0.26
		Replica	5.7	7.1	26.2 ± 0.85
Ginkgo Biloba	all	Leaf	7.4	9.1	42.4 ± 0.6
	all	Replica	8.8	10.4	36.4 ± 1.1

**Table 3:** Solid fraction and Wenzel parameter of each scales of replicas.

Surface	Scale	Solid Fraction $\phi_{S}$	Wenzel Parameter *r*
Phyllostachys replica	Ridge Lines	$\phi_{RL}$ = 0.6	$r_{RL}$ = 1.006
	Oriented Bumps	$\phi_{OB}$ = 0.08	$r_{OB}<$ 1.361
	Simple Bumps	$\phi_{SB}$ = 0.25	$r_{SB}<$ 1.361
Sasa replica	Ridge Lines	$\phi_{RL}$ = 0.55	$r_{RL}$ = 1.007
	Oriented Bumps	$\phi_{OB}$ = 0.68	$r_{OB}<$ 1.260
	Simple Bumps	$\phi_{SB}$ = 0.25	$r_{SB}<$ 1.260
Ginkgo replica	All	ϕ = 0.29	*r* = 1.36

**Table 4:** Predicted contact angle of the wetting configuration.

Surface	Wetting Configuration	Predicted Contact Angle (${}^{\circ }$)	Experimental Contact Angle (${}^{\circ }$)
Phyllostachys replica	1	166	133–135
	2	150	
	3	133	
	4	162	
	5	142	
	6	121	
Sasa replica	1	139	134–136
	2	131	
	3	133	
	4	124	
	5	113	
	6	119	
Ginkgo replica	1	144	131
	6	121	

## Data Availability

All data are available within the manuscript.
